# Stable Panoramic Views Facilitate Snap-Shot Like Memories for Spatial Reorientation in Homing Pigeons

**DOI:** 10.1371/journal.pone.0022657

**Published:** 2011-07-27

**Authors:** Tommaso Pecchia, Anna Gagliardo, Giorgio Vallortigara

**Affiliations:** 1 Centre for Mind/Brain Sciences, University of Trento, Rovereto, Italy; 2 Department of Biology, University of Pisa, Pisa, Italy; University of Arizona, United States of America

## Abstract

Following spatial disorientation, animals can reorient themselves by relying on geometric cues (metric and sense) specified both by the macroscopic surface layout of an enclosed space and prominent visual landmarks in arrays. Whether spatial reorientation in arrays of landmarks is based on explicit representation of the geometric cues is a matter of debate. Here we trained homing pigeons (*Columba livia*) to locate a food-reward in a rectangular array of four identical or differently coloured pipes provided with four openings, only one of which allowed the birds to have access to the reward. Pigeons were trained either with a stable or a variable position of the opening on pipes, so that they could view the array either from the same or a variable perspective. Explicit mapping of configural geometry would predict successful reorientation irrespective of access condition. In contrast, we found that a stable view of the array facilitated spatial learning in homing pigeons, likely through the formation of snapshot-like memories.

## Introduction

Following spatial disorientation, animals can reorient themselves according to geometric cues (metric and sense) specified by the macroscopic surface layout of an enclosed space [1], [2]. Although research (particularly in humans) focussed on reorientation in enclosed spaces [3], recent evidence suggest that animals can also learn to reorient according to geometric cues in arrays of freestanding objects [4]–[6]. This raises the issue of whether re-orientation in arrays of freestanding objects relies on explicit representation of geometric cues (and see for similar concerns about extended surfaces [7]).

Behavioural studies on insects suggest that efficient spatial reorientation can rely on purely egocentered (snap-shot like) memories of the visual scene [8]. The view-matching approach to navigation, pioneered by Cartwright and Collett to describe landmark's use in bees [9], is based on the assumption that movement in space could be immediately derived by comparing specific contents of panoramic views (snapshot) between the target and the current location, until a minimal mismatch is found. Assuming a common directional reference across snapshots, Cartwright and Collett showed that a view-matching algorithm based on both the angular distribution and extension of visual cues in the scene could mimic the spatial behaviour of bees in a laboratory task [9]. Also in a vertebrate species, the domestic chick, it has been recently suggested the use of a view-based strategy, rather than an explicit representation of geometry, for spatial reorientation in an array of landmarks [6]. However due to the complexity of spatial cognition in birds and to robust evidences of geometric representation of environments [1], [10], the hypothesis of a purely view-based strategy in learning spatial features used for re-orientation deserves further investigations.

Here we tested the role of stable panoramic-views of the surrounding in reorientation by homing pigeons in a rectangular array of landmarks. The birds were exposed either to a stable or a variable view of the array during training, and we tested whether this affected spatial learning. If spatial re-orientation depends on an explicit representation of the configural geometry, we expect to observe that performances do not depend on the stability of the view of the surroundings. Alternatively, if a stable view of the surroundings is critical for spatial learning, we expect to observe poorer performances in birds trained with a variable view of the array.

## Methods

### Ethical disclaimer

The experiment was conducted according to the specifications of the Italian law for the prevention of cruelty to animals and has been approved by the Ethical Committee of the University of Pisa (C.A.S.A.), with the permit number 4886-2011.

### Subjects and housing

Experiments took place at the Arnino field station of the Department of Biology of the University of Pisa. Thirty-four unsexed adult pigeons (*Columba livia*) with extensive homing experience were used in the experiment. They were housed in a loft where they received water and grit ad libitum. The food was provided inside two identical PVC pipes in a position which was changed everyday. These pipes (Ø 25 cm, 55 cm height) presented 6 circular openings (Ø 4.4 cm). Three of these openings allowed access to the food, while the others were blocked by a transparent screen. Before the beginning of the experiment the pigeons were food-deprived and then maintained at 80% of their free-feeding body weight. They continued to receive water and grit ad libitum in the loft, while the food was delivered only in the experimental apparatus.

### Experimental set-up

**Figure 1:**
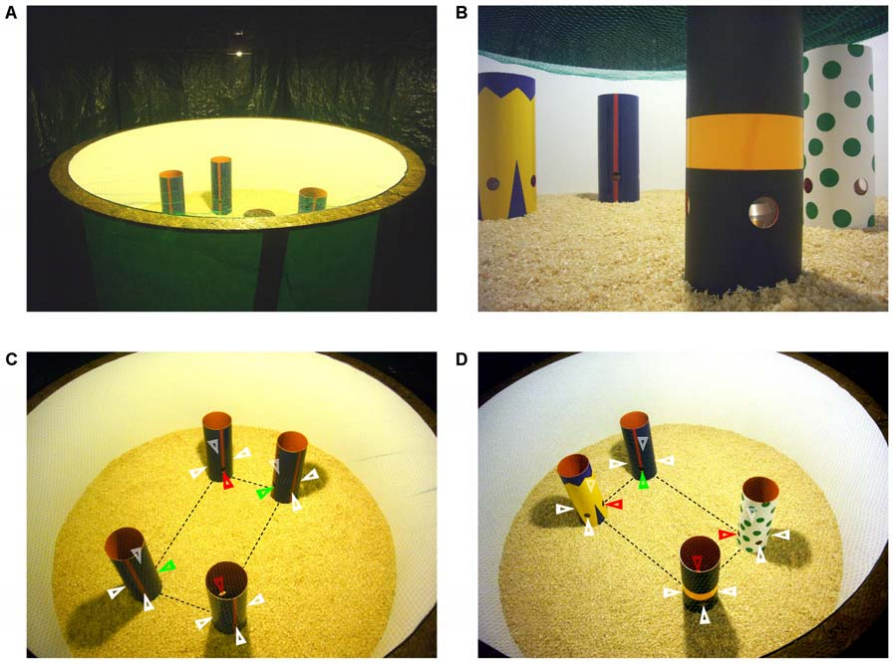
Experimental set-up. Panel A) External view of the apparatus. The dark green plastic curtain surrounded the arena at a distance of approximately 60 centimetres. The arena was constructed from one fibreglass panel covered by a homogeneous white masking tape and it was mounted on a wooden structure. Panel B) A inner view of the arena taken from the vicinity of the wall from the view-point of the pigeon. The circular openings on pipes in the distinctive pipes array and the inner feeders are visible. Panel C) and D) A survey picture of the arena in the identical pipes array (IPA) and in the distinctive pipes array (DPA), respectively. The arrangement of the accessible openings (rewarded pipes: green arrows; unrewarded pipes: red arrows) and the blocked openings (white arrows) for a given training session is schematically represented.

The experimental apparatus consisted of a circular arena (Ø 230 cm; 88 cm height), the floor of which was covered with sawdust (15 cm in depth). A bird-net on the top of the arena prevented the pigeons from flying out. A dark green plastic curtain both surrounded the apparatus and covered the ceiling of the experimental room. A light bulb (180W) hung above the centre illuminating the arena (see Figure 1A).

### Pre-training procedure

The pigeons were subjected to a pre-training procedure as following. Initially, each pigeon was introduced inside the arena with one pipe (Ø 20 cm ×60 cm height) provided with four circular openings (Ø 4.4 cm) spaced 90° with respect to each other and aligned at the same height from the floor. The pipe(s) used for the pre-training was the same as those used for the training phase. When the birds showed that they were able to feed from that pipe, three of the pipes' openings were blocked with a transparent screen, so that the food was accessible only through one hole on each pipe. In the third pre-training phase, a rectangular array (114 cm ×54 cm) of four rewarded pipes with a single accessible opening was presented to the pigeons. After a pigeon gained the food from all the pipes in ten consecutive trials, the training phase started.

### Training procedure

The pigeons were trained to locate the rewarded pipe at specific location(s) in the rectangular array. In particular, two different groups of pigeons were trained in two different pipes arrays in order to investigate the contribution of featural and geometric cues for spatial re-orientation (see Figure 1C, 1D): A) Identical Pipes Array (IPA n = 18 pigeons). Four indistinguishable pipes were presented to the pigeons. The rewarded pipes occupied geometrically equivalent locations in the array (same diagonal). The rewarded diagonal was maintained the same for the same pigeon, but it was changed across pigeons. B) Distinctive Pipes Array (DPA n = 16 pigeons). The pipes were differently coloured and the reward was hidden inside one pipe only. The position of each differently coloured pipe in the array was maintained constant for the same pigeon, but it was changed across pigeons.

In both the experimental settings, one of the openings on each pipe was aligned with the bisectric line of each corner in the array. Only one of the openings on each pipe allowed the pigeons to access the pipes contents. The remaining openings were blocked by a transparent screen as previously described. The positions of the accessible openings were arranged symmetrically in the array within a training session.

**Figure 2:**
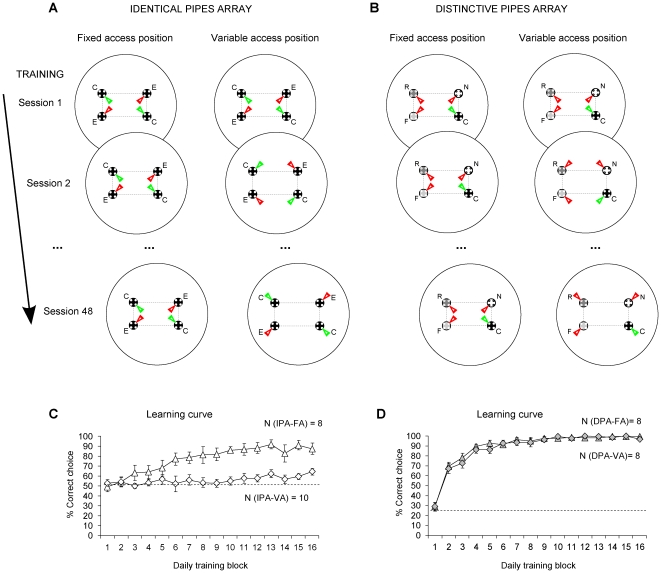
Training. Schematic representation of the accessible openings in the IPA (Panel A) and in the DPA (Panel B) across the training sessions in the FA (left columns) and the VA condition (right columns). For convenience, the array is represented with the same orientation in all panels, but it was rotated between the training sessions. The position of the accessible openings in the FA conditions as well as the rewarded pipes in the two types array are represented here at one position only, but they were changed across pigeons. Bottom Panels: Mean percentage of the correct choices (± SEM) during the training in the identical pipes' array (Panel C: ▵  =  IPA-FA; ⋄  =  IPA-VA) and in the distinctive pipes' array (Panel D: ▴  =  DPA-FA; ⧫  =  DPA-VA).

Both IPA and DPA groups were divided into two subgroups: i) Fixed Access condition (FA). The position of the accessible opening was stable over training sessions for each pigeon, but varied across pigeons; ii) Variable Access condition (VA). The position of the accessible opening was changed between sessions and it was balanced over the total number of sessions for each pigeon. The use of a FA versus VA position to the feeders was intended to force the pigeons to experience either a stable or a variable view of the array at reward. Since the landmarks were presented with four openings, however, the arena retained its visual symmetry in both access condition (see Figure 2A, 2B).

The pigeons were given 3 training sessions of 6 trials each per day for 16 days with one day interruption after 6 days. Within a session each pigeon was released twice from the centre of the arena and once from each side of the array near the wall of the arena, facing different directions and following a pseudo-random order. A choice was scored when the pigeon inserted its beak through an accessible opening. The bird was allowed to consume the reward in the case of a correct choice. Only the first choice in every trial was considered for the analysis. After two consecutive errors within a session, the pigeons were given the opportunity to find the rewarded pipe. Between two consecutive trials, the pigeons were put inside an opaque plastic box (27 cm width ×33 cm length ×22 cm height) located outside from the arena, where they were slowly rotated to prevent them from reorienting on the basis of inertial cues. The array was rigidly rotated (45°, 90°, 135°, 180°, 225°, 270°, 315°, 360°) on its centre between sessions to rule out the use of the magnetic compass, and balancing the orientations over the total number of sessions for each pigeon. After the training each group was subjected to different test conditions. A choice at test was scored following the same criterion as during training.

### Tests in the identical pipes array (IPA)

The IPA-FA pigeons received two test sessions on separate days in the array of four indistinguishable pipes, in order to test the birds' memory retention and their ability to generalize the correct sites from novel view-points. The position of the accessible openings was the same as in the training (fixed access position) in one test session, and it was rotated by 180° with respect to the training (rotated access position) in the other session. The test order was changed across pigeons.

The IPA-VA pigeons received a single test session in the same array as in the training. One of the four positions for the accessible opening used during training was chosen and kept constant for each pigeon, changing the choice of the opening across pigeons. The position of the accessible opening was opposite to the last experienced during training.

### Tests in the distinctive pipes array (DPA)

Both DPA-FA and DPA-VA birds received two test sessions on separate days. One test session was carried out in the rectangular array composed by four indistinguishable pipes of the type reinforced during the training, in order to assess a possible incidental learning of the configural geometry. DPA-FA birds were tested with the same position of the accessible opening on pipes, as in the training. For the DPA-VA birds, the position of the accessible opening was opposite to the last experienced during training and it was kept constant for each pigeon, though it was changed across pigeons. The other test session was carried out in the distinctive pipes' array. The DPA-FA birds were tested with a new position of the accessible opening, which was at 180° with respect to the training position. This test aimed at assessing whether the pigeons could generalize the correct feature from a novel view-point. For the DPA-VA birds one position of the accessible opening was chosen and it remained the same within the session for each bird, changing the choice across birds. The position of the accessible opening was opposite to the last experienced during training. This test aimed at assessing the impact of a variable training condition on featural information retrieval. The test session consisted of ten unrewarded trials. The birds received a short retraining (ten rewarded trials) between the tests session in order to avoid extinction.

A two way repeated measure analysis of variance (Two way RM ANOVA), with the rewarded diagonal and access condition as the between-subjects factors and the mean percentage of the correct choices in each block of three daily sessions as a within-subjects factor, was used to examine the effect of the access conditions (fixed- vs variable- access) on learning. Paired sample t-test was used to examine whether test choices were distributed in the array according to a geometric criterion. Independent sample t-test was used to compare the pigeons' performances between independent test conditions.

## Results

In the identical pipes' array, the analysis of variance revealed a significant main effect of both training blocks (F(15,210)  = 8.526, p = 0.000, η_p_^2^ = 0.378) and access conditions (F(1,14)  = 19.306, p = 0.001, η_p_^2^ = 0.580). A significant interaction between access condition (variable or fixed) and training blocks was also revealed (F(15,210)  = 4.220, p = 0.001, η_p_^2^ = 0.232), indicating that the learning curves' slope differed significantly between IPA-FA and IPA-VA. In fact, only IPA-FA pigeons learnt to locate the reward (Simple effect of training block: IPA-FA: F(15,90)  = 9.666, p = 0.000, η_p_^2^ = 0.617; IPA-VA: F(15,120)  = 1.189, p = 0.334, η_p_^2^ = 0.129) (see Figure 2C).

In the distinctive pipes' array, both the DPA-FA and DPA-VA pigeons learned to locate the rewarded pipe (main effect of training block: F(15,180)  = 104.987, p = 0.000, η_p_^2^ = 0.897; main effect of access condition: F(15,180)  = 0.319, p = 0.583, η_p_^2^ = 0.026; interaction between training block and access condition: F(15,180)  = 0.481, p = 0.748, η_p_^2^ = 0.039) (see Figure 2D).

**Figure 3:**
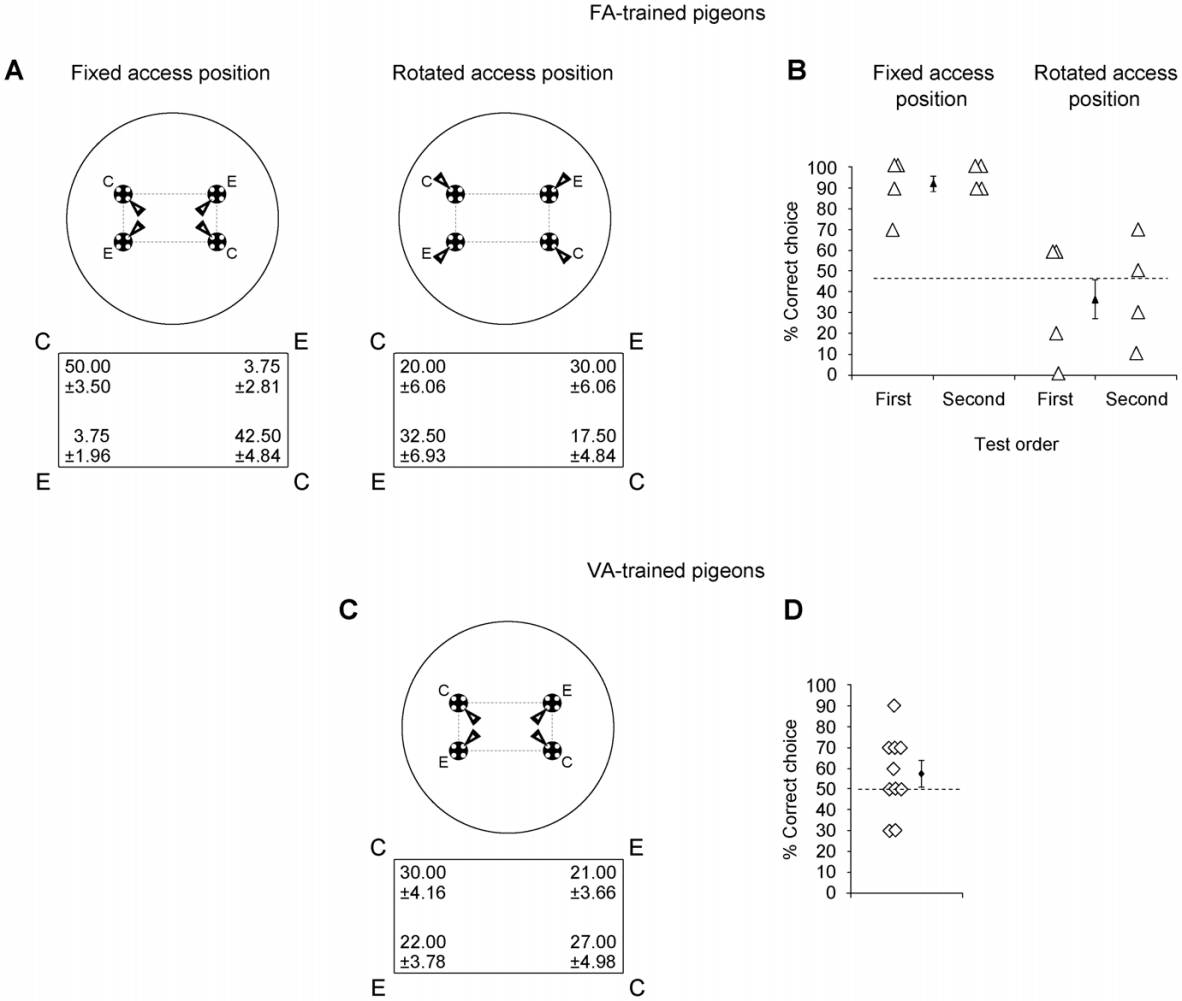
Pigeons' performances at test after training in the identical pipes array. Schematic representations of both the array and the arrangements of the accessible openings on pipes (black arrows) for each tests condition. IPA-FA pigeons were tested both with familiar position (Panel A, Left) and with a rotated position of the accessible openings on pipes (Panel A, Right). IPA-VA pigeons received a single test session in the same array as in the training (Panel C). The mean percentage of choices (± SEM) directed to the four pipes in the array are reported within the correspondent rectangles. Individual percentage of the geometrically correct choices, together with the mean percentage (± SEM) of the correct choices, for each test's condition are reported in the Panels B) and D).

The IPA-FA tested in the fixed position of openings maintained their performances above the chance level (geometrically correct choices vs geometric errors: t(7)  = 11.613, p = 0.000, Cohen's *f* = 5.776; correct diagonal: t(7)  = 1.070, p = 0.320, Cohen's *f*  =  −0.473; uncorrect diagonal: t(7)  = 0, p = 1, Cohen's *f* = 0). The same birds failed to reorient in the rotation position test (geometrically correct choices vs geometric errors: t(7)  =  −1.357, p = 0.217, Cohen's *f*  =  −0.657) (see Figure 3A, 3B). The IPA-VA pigeons failed to locate the geometrically correct pipes in the array (geometrically correct choices vs geometric errors: t(9)  = 1.172, p = 0.271, Cohen's *f* = 0.515) (see Figure 3C, 3D). The mean percentage of the correct choices of IPA-VA and IPA-FA fixed position group differed significantly (t(16)  = 4.757, p = 0.000, Cohen's *d* = 2.427).

**Figure 4:**
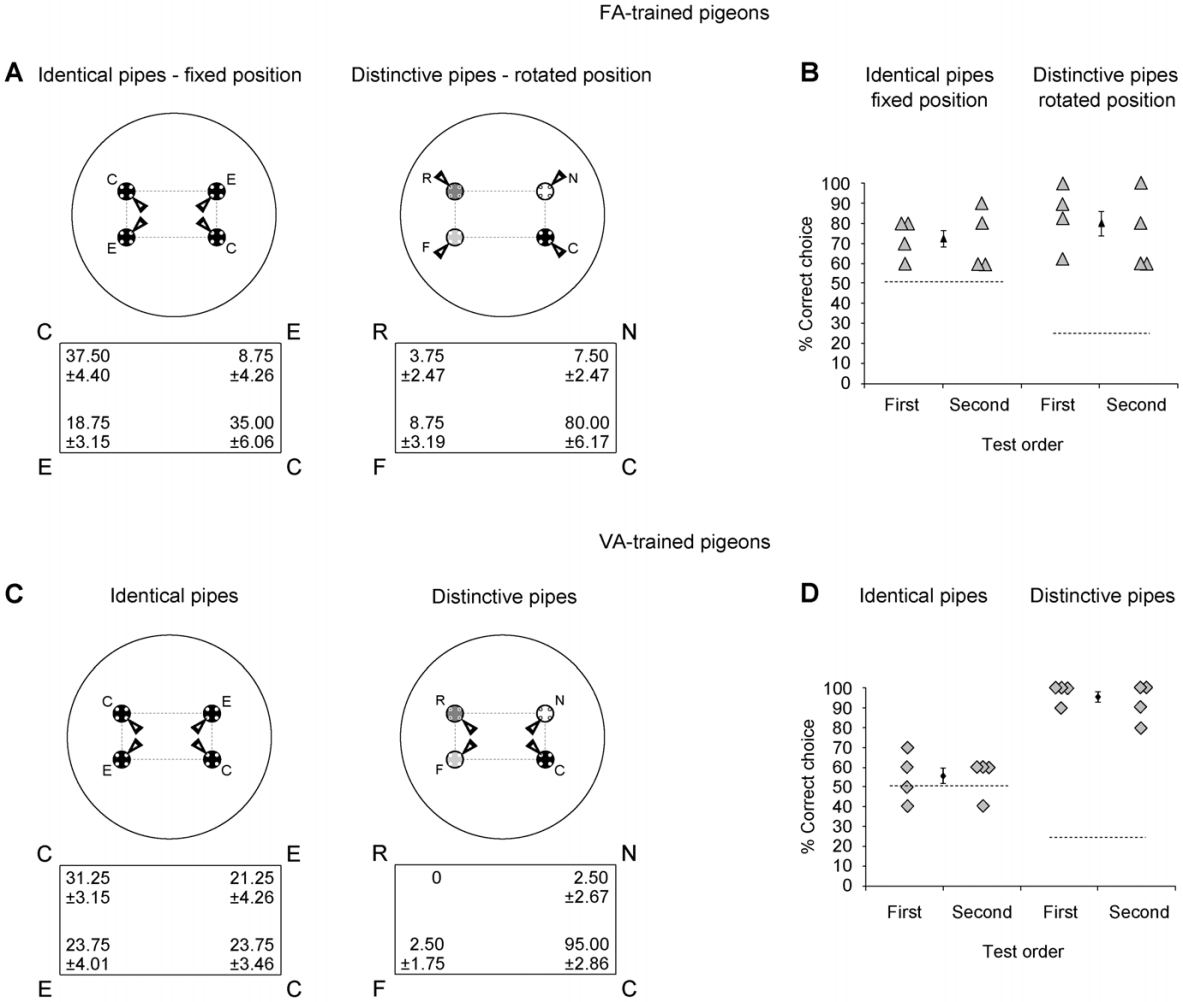
Pigeons' performances at test after training in the distinctive pipes array. Schematic representations of the array and the arrangements of the accessible openings on pipes (black arrows) for each test's condition. DPA-FA pigeons were tested both in the IPA, with a familiar position of the accessible openings on pipes (Panel A, Left), and in the DPA, with a position of the accessible openings which was rotated with respect to the training (Panel A, Right). DPA-VA pigeons were tested both in the IPA (Panel B, Left) and in the DPA (Panel C, Right) with the position of the accessible openings on pipes that was rotated with respect to the last training session. The mean percentage of choices (± SEM) directed to the four pipes are reported within the correspondent rectangles. Individual percentage of the correct choices, together with the mean percentage (± SEM) of the correct choices, for each tests condition are reported in the Panels B) and D).

The DPA-FA pigeons retrieved the configural geometry when tested in an array of four indistinguishable pipes with a familiar accessible opening (geometrically correct choices vs geometric errors: t(7)  = 5.463, p = 0.001, Cohen's *f* = 2.713; correct diagonal: t(7)  =  −0.277, p = 0.790, Cohen's *f*  =  −0.125; uncorrect diagonal: t(7)  = 1.764, p = 0,121, Cohen's *f* = 0.712). The DPA-FA pigeons were also able to locate the correct pipe in the distinctive pipes array when the accessible opening was in the opposite position with respect to the training (correct choice: t(7)  = 9.537, p = 0.000, Cohen's *f* = 2.567) (see Figure 4A, 4B).

Also the DPA-VA pigeons successfully located the correct pipe in the distinctive pipes array (correct choices: t(7)  = 26.192, p = 0.000, Cohen's *f* = 8.395), and even more accurately than the DPA-FA (t(14)  = 2.356, p = 0.040, Cohen's *d* = 1.259). By contrast, the DPA-VA birds failed to retrieve the geometric cues when tested in the identical pipes' array (geometrically correct choices vs geometric errors: t(7)  = 1.323, p = 0.227, Cohen's *f* = 0.658) (see Figure 4C, 4D). Therefore DPA-FA birds turned out to choose the geometrically correct pipes significantly more often than DPA-VA birds (t(14)  = 3.130, p = 0.007, Cohen's *d* = 1.570). The DPA-FA birds, however, made more geometric errors than IPA-FA pigeons (t(14)  = 3.630, p = 0.003, Cohen's *d* = 1.940), suggesting the occurrence of cue competition between geometric and non-geometric cues.

## Discussion

The present work aimed at investigating the abilities of homing pigeons to re-orient in an array of freestanding objects. The procedure parallels previous laboratory studies of spatial re-orientation in rectangular shaped enclosures [1], except that both geometric and non-geometric cues were provided by four proximal landmarks arranged in a rectangular shaped array rather then by the shape of the three-dimensional surface layout of the arena. Our results confirmed previous findings indicating that non-human animals (rats [4], Clark's nutcrackers [5] and domestic chicks [6]) are able to reorient, at least under certain condition, according to configural geometry in a rectangular array of landmarks [10].

In order to clarify the nature of the underlying spatial representation, we tested the impact of stable panoramic views at reward on the pigeons' performances. The birds were trained both in a rectangular array of four distinctively (DPA) coloured pipes and in a rectangular array of four indistinguishable (IPA) pipes. In both the pipes arrays, the pigeons were trained either with a fixed (FA) or a variable (VA) position of opening allowing the access to the food reward. In all of the experiments, the pigeons were released in the arena from different positions and were disoriented between trials in order to prevent them from relying on inertial guidance cues to re-orient. Our results showed that the IPA-FA pigeons learnt to reorient in the array and failed to generalize the geometrically correct landmarks from a novel view-point; the IPA-VA pigeons were not able to solve the task at all; the DPA trained pigeons retrieved the configural geometry when tested in an array of four indistinguishable pipes provided that the position of the accessible opening was maintained the same throughout the training and the test sessions. It could be concluded therefore that a stable panoramic view at reward, rather than the configural geometry per se, re-orient in a homogeneous array of landmarks.

On the other hand, pigeons learnt to reorient on the basis of the featural cues when trained in an array of visually distinctive cylinders, regardless the access condition. This suggests that geometric-based learning might be dissociated from featural-based learning. However, an egocentered strategy cannot be excluded even for featural based learning. In fact, the DPA-VA pigeons might have encoded progressively during training invariant visual information about the rewarded feature from unstable panoramic-views. Although the DPA-FA pigeons generalized the correct coloured pipe at test from a novel access position, they made more featural errors than DPA-VA pigeons tested in the distinctive pipes' array, indicating that any changes in the panoramic-views deteriorated the pigeons' performances. In other words, birds exposed during training to a fixed panoramic-view (DPA-FA) learnt the feature of the rewarded pipe in conjunction with other kinds of stable information, including a particular arrangement of the pipes in the scene. In the rotation test these information were set in conflict and produced lower accuracy in the choice of the DPA-FA birds. By contrast, the birds trained to get the reward from a distinctive coloured pipe, but with a variable perspective of the array (DPA-VA), were forced to rely exclusively on the featural cues characterizing that pipe to solve the task. This condition produced more correct test choices in this group. This finding suggests a unitary learning process for spatial re-orientation based on both featural and geometric cues. In agreement with associative learning theories [11], featural and geometrical stimuli might have therefore competed with each other for taking control over the animal responses. Evidence of such cue-competition occurred indeed. In fact, the IPA-FA pigeons were more accurate than the DPA-FA pigeons when tested in the identical pipe's array with a fixed access position, showing that training experience with a distinctive coloured pipes array attenuated geometrical learning.

Since both geometric and featural information were apparently encoded in egocentered coordinates, the results are consistent with a global matching strategy (i.e. a pixel-by-pixel matching) of spatial reorientation, based on the formation of snapshot-like memories of the surroundings [8]. Indirect support for the use of snapshot-like memories in navigation by pigeons has been occasionally reported in previous studies in the field [12], [13]. Pre-exposure of a particular sector of the familiar landscape already before release has proven to increase homing speed in homing pigeons [12]. It has been reported that homing pigeons tend to recapitulate their route when released repeatedly from the same site [13]. Considering the results reported here, it could be speculated that route loyalty might facilitate learning of stable panoramic views along the homing flight, providing the birds with a reliable spatial representation against changes in the featural layout. A memory trace of stable panoramic view of the surrounding may provide, at least under certain condition, advantages analogous to explicit geometric computation in spatial tasks [14]. Likewise, domestic chicks take advantage in both a visual discrimination task [15] and a visuo-spatial re-orientation task [6] from perceiving the stimuli in the scene from highly stereotyped vantage points. Caution is suggested, however, in considering these findings as conclusive against flexible use of landmarks in avian species, particularly in the wild.

The contents of panoramic views used by birds to re-orient still remain largely unexplored. Although our findings could be accounted for on the basis of a global-matching strategy for spatial reorientation, it could be hypothesized that the view-matching process in avian species operates on a subset of information contents from panoramic views rather than on a pixel-by-pixel matching. It could be that pigeons extract object-related information, in particular the featural cues characterizing landmarks, and use that information either individually or in association with a genuine geometric discrimination sense. In the former case, featural information characterizing the objects in the scene would be treated as beacons to direct the pigeons' choices in the environment. In the latter case, featural cues characterizing multiple objects would be combined in a relational representation of the scene, anchored on an egocentric frame of reference (“there is a yellow landmark in the scene and it is on the left of the rewarded site”: see Figure 1D). On the basis of the results at hand, it is not possible to conclusively determine which strategy was used by pigeons to re-orient in our task. Further experiment are needed to clarify this issue. Nevertheless, our findings clearly indicate that homing pigeons reorient in an array of landmarks in an arena on the basis of a purely egocentered representation of the visual scene.
